# Supplementation of Methionine Dipeptide Enhances the Milking Performance of Lactating Dairy Cows

**DOI:** 10.3390/ani14091339

**Published:** 2024-04-29

**Authors:** Xiaoshi Wei, Ning Han, Hongyun Liu

**Affiliations:** 1 College of Animal Science, Zhejiang University, Hangzhou 310058, China; deardoris163@163.com (X.W.); hanning@zju.edu.cn (N.H.); 2 College of Animal Science and Technology & College of Veterinary Medicine, Zhejiang A&F University, Hangzhou 311300, China

**Keywords:** methionine dipeptide, milk protein, rumen fermentation, bacterial composition, dairy cows

## Abstract

**Simple Summary:**

Methionine dipeptide (Met-Met) could improve milk protein synthesis in bovine epithelia mammary cells and lactating mice, while the effects of Met-Met on lactating dairy cows have not been explored. We hypothesized that supplementation of Met-Met could improve lactation performance of dairy cows. In this study, we elucidated the effects of different forms of Met-Met (rumen-protected and -unprotected) on lactation performance, rumen fermentation and microbiota profile of dairy cows. Results provided the first evidence that Met-Met supplementation can improve lactation performance of dairy cows, and suggested that the rumen-protected form presents greater benefits on milk performance and rumen behavior.

**Abstract:**

Methionine dipeptide (Met-Met) could improve milk protein synthesis in bovine epithelia mammary cells and lactating mice, while the effects of Met-Met on lactation performance, rumen fermentation and microbiota profile in lactating dairy cows have not been explored. For this reason, 60 Chinese lactating Holstein cows were allocated into three treatment groups: control group (CON), 6 g/d methionine dipeptide group (MM), and 6.12 g/d rumen-protected methionine dipeptide group (RPMM). The experiment lasted for 10 weeks to monitor lactation performance, plasma amino acid profile and rumen fermentation parameters and microbiota profile. Results showed that MM increased the energy-corrected milk (ECM), and RPMM increased both milk yield and ECM (*p* < 0.05). The milk protein concentration and yield were increased by MM and RPMM (*p* < 0.05). The rumen fermentation showed that RPMM increased total volatile fatty acids, acetate and valerate concentrations (*p* < 0.05). The relative abundance of *Firmicutes*, including *Succiniclasticum*, *Selenomonas* and *Clostridium_XlVa*, were enriched and the *Prevotella* was decreased by RPMM (*p* < 0.05). In summary, daily supplementing with 6 g of MM or RPMM in lactating dairy cows could improve milk yield and both percentage and yield of milk protein, and RPMM benefited the rumen fermentation and altered the bacterial composition. These results provided the first evidence that Met-Met supplementation can improve lactation performance of dairy cows.

## 1. Introduction

Milk, as the main food for humans, not only provides necessary energy and nutrients but also plays an important role in maintaining health related to the immune system, gut microecology and nutrition balance [1]. Milk protein is a major component in milk, providing amino acids (AAs) and nutritional and bioactive proteins, and also represents the milk quality and economic value. AAs and peptides act as building blocks and key regulators of milk protein. Peptides can make up for the deficiency of AAs for milk protein synthesis [2], and have become popular in research due to effective and energy-saving features. Therefore, the effects of dipeptides on milk protein synthesis and further utilization are worth exploring.

Methionine (Met) is considered as the first limiting AAs of dairy cows in corn–soybean meal-based diets. Supplementation of Met to dairy cows has been proven to be effective to promote lactation performance [3]. Met dipeptide (Met-Met) is a bioactive peptide consisting of two Met residues linked by a peptide bond. Our previous studies in vitro demonstrated that Met-Met promoted αS1-casein synthesis in cultured bovine mammary gland explants [4], and enhanced cell proliferation and β-casein synthesis in bovine mammary epithelia cells (BMECs) [5]. In addition, studies in lactating mice demonstrated that Met-Met supplementation increased αS1-casein production and milk yield compared with the mice fed Met [6], and was proven to be more effective than Met in promoting mammogenesis and lactogenesis [7]. Whether Met-Met could alter the lactation performance of dairy cows has not been reported.

Rumen fermentation is crucial for energy supply of the host, and microbial composition exerts a large effect on milk nutrients [8]. Ruminal microbes might degrade Met-Met, making it unavailable for the animal. Therefore, we hypothesized that supplementation of Met-Met could improve lactation performance of dairy cows, and rumen-protected Met-Met would be more effective. Thus, the objectives of this study are (1) to investigate the effects of Met-Met supplementation on lactation performance, rumen fermentation and microbiota profile; and (2) to compare the similarity and difference of the different supplementing forms of Met-Met, so as to determine which would make a preferred additive.

## 2. Materials and Methods

### 2.1. Animal Ethics Statement

The experiment was approved by the Institutional Animal Care and Use Committee of Zhejiang University (Hangzhou, China), and the experiment procedures were in accordance with the guidelines for animal research.

### 2.2. Animals and Experimental Design

Sixty Chinese Holstein lactating dairy cows were selected based on their similarities in body weight (602 ± 78.5 kg), average milk production (30.32 ± 2.26 kg/d), days in milk (161.4 ± 42.7 d), and parity (2.3 ± 0.8). Cows were randomly allocated into 1 of 3 treatment groups (n = 20 in each): control group (CON, basal diet), Met-Met group (MM, Evonik Industries, Piscataway, NJ, USA), and rumen-protected Met-Met group (RPMM, Hangzhou King Techina Feed Co., Ltd., Hangzhou, China). The RPMM was provided as 17 g/d as the RPMM product contains 50% Met-Met, the rumen bypass rate was 90% and the small intestinal release rate was 80%. Therefore, the Met-Met supplementation of CON, MM and RPMM groups were 0, 6 and 6.12 g/d, respectively. The basal diet (Table 1) was offered as total mixed ration (TMR) to allow for ad libitum consumption at 06:30, 14:30 and 22:30 h. Before feeding, the supplements were top-dressed small amount of fresh TMR and supplemented in front of each cow for individual consumption, ensuring all supplements were consumed completely. Cows were milked with equipment 3 times a day and had free access to feed and water. The experiment lasted for 10 weeks, of which the first 2 weeks were the adaptation period.

### 2.3. Sampling and Analyses

Feed composition and intake: TMR samples offered and the residuals were recorded and collected weekly. Samples were dried at 65 °C for 72 h and ground through a 1 mm sieve before analysis. The dry matter intake (DMI) was calculated by TMR offered and residual. The CP (GB/T6432-2018 [10]), NDF (GB/T20806-2006 [11]), ADF (NY/T1459-2007 [12]), EE (GB/T6433-2006 [13]), Ash (GB/T6438-2007 [14]), Ca (GB/T6436-2018 [15]) and P (GB/T 6437-2018 [16]) were determined; the NFC was calculated and the net energy for lactation was calculated based on the Ministry of Agriculture of China recommendations (NY/T 34-2004 [9]). The ingredients and chemical compositions of the diet are listed in Table 1.

Lactation performance: Milk yield was recorded at each milking using digital flowmeter. Milk samples of each cow were collected weekly at 3 consecutive milking and mixed proportionally (4:3:3). The milk preservative (potassium dichromate, D&F Control Systems, USA) was added for analyses of milk fat, protein, lactose, milk urea nitrogen (MUN) and milk somatic cell count (SCC) using infrared spectroscopy (MilkoScan; Foss Electric, Hillerød, Denmark). The energy-corrected milk (ECM) was calculated using the following formula: ECM = 12.55 × fat yield (kg/d) + 7.39 × protein yield (kg/d) + 5.34 × lactose yield (kg/d).

Plasma amino acid concentration: At the end of the experiment, ten cows were randomly selected from every group, and blood samples (10 mL) were collected from their coccygeal vein by venipuncture into heparinized tubes before morning feeding. Samples were centrifuged immediately at 3000× *g* for 15 min, and the supernatant was collected. Aliquots of the plasma samples were analyzed for plasma AAs by an auto-analyzer (Hitachi L-8900 amino acid analyzer, Tokyo, Japan). 

Rumen fluid and volatile fatty acid: Ten rumen fluid samples were collected from the cows selected at the end of the experiment. About 50 mL of rumen fluid was collected approximately 3 h after morning feeding using an oral rumen fluid sampler. To avoid saliva contamination, the first 150–200 mL of ruminal content was discarded. The pH was measured immediately, and the samples were divided to 3 aliquots. A total volume of 20 μL of 85% orthophosphoric acid was added to 1 mL of rumen fluid to avoid dissociation of acid, and the volatile fatty acid (VFA) was analyzed using gas chromatography according to a previous methodology [17]. Ammonia nitrogen was determined according to Hu et al. [18]. About 2 mL of rumen fluid sample was placed into a 5 mL sterile frozen storage tube and stored at −80 °C for further analyses of microbiota.

### 2.4. Ruminal Microbiota Profile

DNA Extraction and 16S rRNA gene sequencing: Ten rumen fluid samples (n = 10 in each) were randomly selected from every group and prepared for microbiota analysis. Microbial community genomic DNA was extracted from rumen content using the FastDNA^®^ Spin Kit for Soil (MP Biomedicals, Santa Ana, CA, USA, USCAT NO.116560–200, Omega Bio-tek, Norcross, GA, USA). The extracted DNA was checked using agarose gel and the concentration was measured using a NanoDrop 2000 spectrophotometer (Thermo Scientific, Wilmington, DE, USA). Primers 338F/806R (ACTCCTACGGGAGGCAGCAG/GGACTACHVGGGTWTCTAAT) were used to amplify the V3–V4 hypervariable regions by PCR thermocycler (ABI, Los Angeles, CA, USA). The PCR amplification was performed as follows: 95 °C for 5 min; 30 cycles of denaturing at 95 °C for 15 s, 55 °C for 30 s, and 72 °C for 30 s; a single extension at 72 °C for 10 min, and ending at 4 °C. The PCR reactions were performed in triplicate in a 20 μL mixture. The PCR product was extracted from agarose gel, purified, and quantified by Agilent 2100 Bioanalyzer (Agilent, Santa Clara, CA, USA). The purified amplicons were pooled at equimolar ratio and paired-end sequenced on an Illumina HiSeq platform at BGI Genomics Co., Ltd. (Shenzhen, China).

Processing of sequencing data: Analysis of sequencing data Raw fastq files were quality-filtered by Trimmomatic and merged by FLASH (v1.2.11) [19]. Operational taxonomic units (OTUs) were clustered with a 97% similarity cutoff using UPARSE (version 7.1). The taxonomy of each 16S rRNA gene sequence was analyzed using the RDP Classifier algorithm (http://rdp.cme.msu.edu/) (accessed on 30 September 2016) against the database. The α diversity and β diversity analyses were performed using Mothur (version 1.31.2) [20] and QIIME (version 1.80) [21], respectively. The microbiota composition at different levels was determined based on tax_summary and R package (version 3.3.1), and the difference between the groups was analyzed using a one-way ANOVA and Tukey’s test. Linear discriminant analysis effect size (LEfSe) was conducted to screen differentially abundant bacteria with an LDA score of >2.0. Bacterial genera with relative abundances >0.10% in at least 60% of the cows within each group were used for further comparative analyses. The correlation analysis was conducted using Spearman correlation analysis with R (version 4.02) and visualized in Heat map diagram, in which the correlation significance was indicated by asterisk.

### 2.5. Data Analysis

Data statistics were performed by a MIXED procedure of SAS statistics (version 9.4). The repeated procedure was used for variables repeatedly measured over time, such as the DMI and lactation performance. The data on plasma parameters, rumen fermentation and bacterial composition were analyzed using the linear model without repeated measures. In addition, the one-way ANOVA was conducted to compare the differences among 3 groups on energy-corrected milk and milk protein composition at a given time point. The statistical differences were declared significant at *p* ≤ 0.05 and the tendencies were considered at 0.05 < *p* ≤ 0.10.

## 3. Results

### 3.1. DMI and Lactation Performance

The overall effects of treatments are shown in Table 2. The milk yield was increased in the RPMM group compared with CON and MM groups (*p* = 0.01). The ECM was increased in both MM and RPMM groups, with a greater effect observed in the RPMM group (*p* = 0.04). The milk protein yield and percentage were higher in both MM and RPMM groups compared with CON (*p* = 0.03 and 0.04, respectively). Except for DMI, milk fat yield and MUN, there were weekly effects (*p* < 0.05). There were significant changes for treat × week interaction on milk protein yield and percentage (*p* < 0.05).

**Figure 1:**
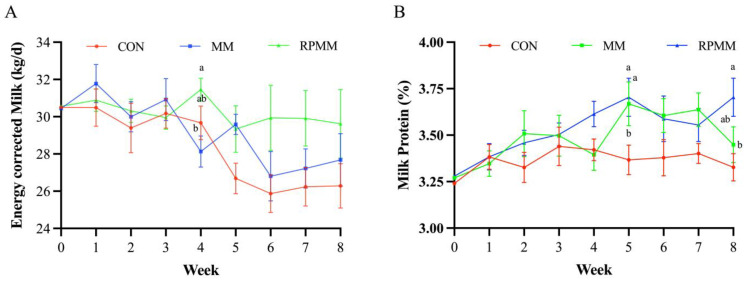
Effects of methionine dipeptide supplementation on energy-corrected milk (**A**) and milk protein percentage (**B**) of lactating dairy cows. Energy-corrected milk = 12.55 × fat yield (kg/d) + 7.39 × protein yield (kg/d) + 5.34 × lactose yield (kg/d), from Orth. CON = control group; MM = methionine dipeptide group; RPMM = rumen-protected methionine dipeptide group. ^a,b^ Means within a row with different superscripts differ (*p* < 0.05).

In addition, Figure 1 shows the weekly changes of ECM and milk protein percentage of dairy cows. At week 4, the ECM in the RPMM group was higher than that in the CON group (*p* < 0.05). The milk protein percentage at week 5 was increased in both MM and RPMM groups compared with CON, and the RPMM increased the milk protein percentage compared with CON at week 8 (*p* < 0.05). Met-met supplementation increased 4.2% milk protein composition (*p* < 0.01), without effect on other components of milk (*p* ≥ 0.16).

### 3.2. Plasma Amino Acid Concentrations

The plasma AA concentrations are presented in Table 3. There were no effects of Met-Met supplementation on plasma AA concentration (*p* > 0.05).

### 3.3. Fermentation Parameters

There were no effects on ruminal pH and NH_3_-N concentration. Total VFA and acetate concentrations were increased in the RPMM group compared to CON and MM groups (*p* < 0.05, Table 4), but the ratio of acetate to propionate was not affected. The MM resulted in a decreased butyrate concentration as compared to CON and RPMM groups (*p* < 0.05). Moreover, the valerate concentration was increased in the RPMM group compared to the CON (*p* < 0.05).

### 3.4. Rumen Bacterial Composition and Correlation

**Figure 2:**
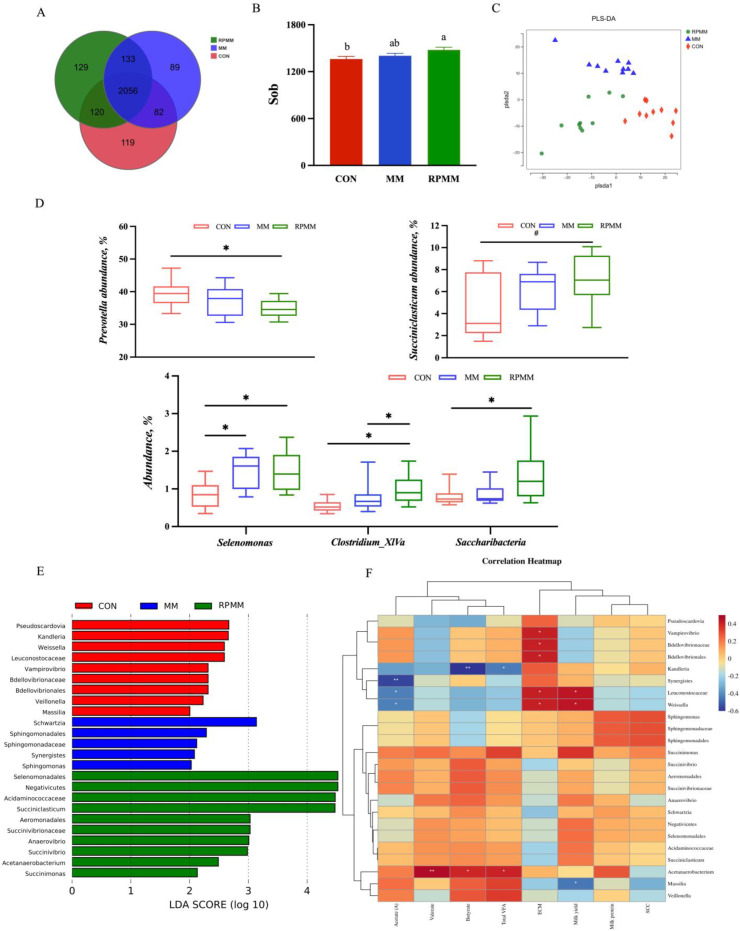
The rumen microbiota profiles of lactating dairy cows supplemented methionine dipeptide. (**A**) Venn diagram presenting the operational taxonomic units (OTUs) from each group. (**B**) α diversity of bacteria. ^a,b^ Means with different superscripts differ (*p* < 0.05). (**C**) β diversity shown in a principal component analysis (PCA) scatterplot. (**D**) A box plot of the significant genera among groups. * 0.01 < *p* ≤ 0.05, # 0.05 ≤ *p* < 0.1. (**E**) Histogram of linear discriminant analysis (LDA) scores representing the taxonomic biomarkers by LDA effect size (LEfSe) analysis. LDA score (log10) >2 suggests the enriched taxa in cases. (**F**) Heatmap diagram of correlations between rumen bacterial and rumen fermentation and lactation performance at genus level. Note: Red represents positively correlated and blue represents negatively correlated. Correlation significance *p*-value is indicated by asterisks. ** *p* ≤ 0.01, * 0.01 < *p* ≤ 0.05.

A total of 2728 OTUs were identified in three groups, among which 2056 OTUs were found in all three groups, accounting for 75.37% of the total OTUs (Figure 2A). The co-expression analysis identified that there were 133 OTUs found in both MM and RPMM groups, 120 OTUs found in both CON and RPMM groups, and 82 OTUs found in both CON and MM groups. The number of OTUs specifically found in the RPMM group, MM group and CON group was 129, 89 and 119, respectively. Compared with CON, the α diversity index Sob was increased in RPMM group (*p* < 0.05, Figure 2B). In addition, the PLS-DA showed clear separations of the rumen bacteriome among the three groups (Figure 2C). 

At the genus level, the relative abundance of *Prevotella* decreased in the RPMM group compared to the CON group (*p* = 0.04, Figure 2D), and the *Succiniclasticum* tended to increase (*p* = 0.065). Both MM and RPMM increased the relative abundance of *Selenomonas* (*p* = 0.01), and the *Clostridium_XlVa* was higher in the RPMM group (*p* = 0.02). The relative abundances of *Saccharibacteria* were increased in the RPMM group compared to CON group (*p* = 0.01). The result of LEfSe showed that enriched bacteria in the CON group were *Proteobacteria (Bdellovibrionales*, *Bdellovibrionaceae*, *Vampirovibrio*, *Massilia)*, *Firmicutes* (*Leuconostocaceae*, *Veillonella*, *Kandleria* and *Weissella*), and *Actinobacteria* (*Pseudoscardovia*) (Figure 2E). In the MM group, *Proteobacteria* (*Sphingomonas*, *Sphingomonadaceae*, *Sphingomonadales*), Firmicutes (*Schwartzia*) and Synergistetes (*Synergistes*) were enriched. Moreover, the *Proteobacteria* (*Succinimonas*, *Succinivibrio*, *Succinivibrionaceae*, *Aeromonadales*) and *Firmicutes* (*Succiniclasticum*, *Acidaminococcaceae*, *Negativicutes*, *Acetanaerobacterium*, *Selenomonadales* and *Anaerovibrio*) were enriched in the RPMM group.

The correlations between rumen bacteria and rumen fermentation and lactation performance were examined (Figure 2F). The concentration of acetate was negatively correlated to *Synergistes*, *Leuconostocaceae* and *Weissella*. The *Acetanaerobacterium* was positively correlated to valerate, butyrate and total VFA concentrations, and *Kandleria* was negatively correlated to butyrate and total VFA concentrations. In addition, both milk yield and ECM were positively correlated to *Leuconostocaceae* and *Weissella*, and ECM was additionally positively correlated to *Bdellovibrionales*, *Bdellovibrionaceae* and *Vampirovibrio*. The milk yield was negatively correlated to *Massilia*.

## 4. Discussion

Supplying sufficient and balanced AAs plays a critical role in improving milk protein concentration and yield of dairy cows [22,23]. Methionine (Met) is considered as the first limiting AAs of dairy cows in corn–soybean meal basal diet, and supplementation of Met product has been widely used to improve the lactation performance [24]. In previous in vitro studies, it has been reported that Met-Met could promote α-s1 casein synthesis in both cultured bovine mammary gland explants and epithelial cells [4,5] and lactating mice [6,7], while there was no report on dairy cows. In this study, we explored the effects of Met-Met supplementation on lactation performance and rumen microbiota profile of dairy cows. 

In this study, the percentage and yield of milk protein were increased in both MM and RPMM. The promotion of milk protein synthesis and secretion would be related to the intracellular substrate availability, cell proliferation and signaling molecules [25]. In addition to AAs, the peptides were found to have been taken up by the mammary glands and utilized for milk protein synthesis, accounting for more than 25% of the milk protein [26]. Usually, some of peptide could be taken up in intact form, and some are hydrolyzed to the corresponding free AAs and then absorbed [5,27]. Previous research has shown that Met-Met increased the uptake of Met, Lys, His, Val, Leu and Phe in vitro, and Phe-Phe promoted the total uptake of Lys, Leu, Ile, Phe and Val [4,28]. Studies have shown that the Met-Met could increase the expression of peptide transporters 2 (PepT2), along with increased gluconeogenic AA concentrations and improved lactation performance [7,29]. In this study, however, we measured the plasma AA concentrations, but no changes were found. Thus, we speculated that Met-Met provided by MM and RPMM would be absorbed directly through small peptide transporters [30]. The uptake of Met-Met through small peptide transporters reduced the competition with AA uptake by AA transporters, supporting the results of increased milk protein percentage and yield in this study.

Moreover, one of other reasons for the increased milk protein percentage and yield would be due to a higher efficiency in the synthesis of proteins and energy saving (transport across the cell membrane) in Met-Met compared to Met. Met-containing peptides have long been shown to be a Met source for protein accretion. The di- and tripeptide-bound Met was proven to have a 15–76% greater efficiency for the synthesis of proteins in lactating mammary tissue of mice, and the different abilities among the peptides might be due to different transport efficiencies across the mammary cell membrane [2]. Met partially replaced by Met-Met improved reproductive performance, increased milk yield and energy production in Met-deficient pregnant mice, which might be mediated by promoting nutrient availability and activating signaling pathway [4,6,7]. It is suggested that the Met-Met supplementation in this study might increase the efficiency of substrate transport and milk protein synthesis. In addition to acting as a substrate for milk protein synthesis and having higher synthesis efficiency, the signaling molecules are also involved in the milk protein synthesis by Met-Met. Collectively, the promotion was mediated by JAK2-STAT5, mTOR and PI3K-AKT signaling pathways [4,5,7]. Further validation on signaling pathways in the mammary gland of dairy cows through biopsy is needed.

Rumen is an important digestive organ for ruminants. The VFA was produced by ruminal symbiotic microbiota, providing around 60–80% of energy requirements. High fermentability carbohydrates is the main source in VFA production. Met-Met has been proven to be more efficient than other nitrogen sources to increase ruminal total VFA concentration and support VFA acetate producer growth [31]. Similarly, we found that RPMM increased the total VFA concentration by 10.4%, suggesting more energy would be provided for the host. It is well known that rumen fermentation affects the milk composition in dairy cows. RPMM increased ruminal acetate, which can directly participate in the synthesis of milk fat [32]. Feeding sodium acetate increased milk fat yield by 90 g/d and concentration by 0.2 percentage units [33]. However, the increased ruminal acetate did not elicit changes in milk fat performance in this study. A similar trend was also found in ruminal valerate, which was conducive to improved growth [34], as the digestibility of DM, CP, NDF and ADF were significantly higher in in vitro fermentation with Met-Met supplementation [31]. Higher concentrations of VFA (total VFA, acetate, butyrate and valerate) which we found in RPMM further suggested that these cows may have higher ruminal fermentation efficiency without decreasing the ruminal pH.

In this study, the rumen microbiota was characterized with Met-Met supplementation. We identified the bacterial alpha diversity indices, and the results revealed a higher richness (Sob and Ace indexes) with RPMM supplementation. Higher richness of rumen microbiota has also been observed in Met-Met fermentation in vitro [31] and in lactating dairy cows with higher ECM supplemented with rumen-protected Met [35]. The *Selenomonas* was a bacterium producing acetate, propionate, valerate, caproate and heptanoate from glucose [36], which was found enriched in both MM and RPMM. The higher abundance of *Selenomonas* was also found in cows having higher milk yield and MP content [37]. In addition, enriched *Selenomonas* could utilize lactate to generate propionate, thus reducing lactate accumulation [38]. Indeed, we did not find changes in pH and propionate. The enriched *Clostridium* strains in RPMM, known as cellulolytic, proteolytic and amylolytic bacteria, possess the potential to synthesize acetate and butyrate. This was in accordance with the increased acetate in RPMM, favoring the improved rumen fermentation by RPMM.

Peptides and AAs are potential nutrients for the growth of ruminal microorganisms that are also able to degrade to ammonia as a consequence, and are thus lost from the rumen [39]. Rumen-degradable peptides supplied improved fermentation efficiency and microbial output, which in turn improved animal performance [40,41]. The feed efficiency of beef cattle was positively associated with ruminal *Succiniclasticum* [42], so were the milk production and milk protein yield [8,43]. Generally, the degradable Met-Met would be more in MM than that in RPMM, while the higher relative abundance of *Succiniclasticum* was presented in cows supplemented with RPMM; the better milking performance and enhanced rumen fermentation also confirmed the role of RPMM in lactating dairy cows. The predominant genus, *Prevotella*, in rumen can utilize starch and protein to produce succinate and acetate. Previous studies have proven that *Prevotella* plays roles in microbial metabolites, including AA, glutathione, starch, sucrose and galactose metabolisms, and CP digestibility [37,44]. We found that the relative abundance of ruminal *Prevotella* was not changed by MM as compared with CON, which was in accordance with the study of Kong et al. [31], and RPMM decreased the *Prevotella.* Moreover, the genus *Acetanaerobacterium* was an enriched bacterium and was found to be positively corrected to valerate, butyrate and total VFA in this study. The outcome of the levels of VFA, bacterial composition and functional difference might contribute to differences in the metabolism of the host, and therefore, the performance.

According to our previous in vitro study, approximately 35% of RPMM used in this study would be degraded and utilized by rumen microbes of dairy cows. Thus, we presumed that the differences of rumen fermentation and microbiota profile between MM and RPMM might be related to the amount of Met-Met degraded and utilized by rumen microbes, or the rumen-protection technology. Moreover, the effects of Met-Met on rumen fermentation and microbiota is limited, and so, more evidence is needed to verify.

## 5. Conclusions

In this study, Met-Met supplementation, as forms of MM and RPMM, both increased the milk yield and milk protein percentage and yield in lactating dairy cows. RPMM improved the rumen fermentation, increased bacterial abundance and changed the composition of rumen microbiota. Our results provided the first evidence that Met-Met supplementation improved the lactation performance of dairy cows. Results suggest that lactating dairy cows supplemented with RPMM improve milk performance and favor better rumen fermentation conditions. More research is warranted to look into the metabolic changes of Met-Met on rumen and host.

## Figures and Tables

**Table 1:** Ingredient and nutrient compositions of basal diet.

Items ^1^	% of DM
Ingredient	
Alfalfa hay	12.56
Oat hay	1.57
Cornsilage	40.57
Cottonseed	4.97
Beet pulp	3.40
High-humidity corn	3.14
Corn meal	4.45
Flaked corn	14.92
Rumen bypass soybean meal	2.09
Soybean meal	5.76
Rapeseed meal	2.09
Fat powder	0.89
Sodium bicarbonate	0.60
Expanded soybean	1.05
Expanded concentrate ^2^	1.94
Nutrient ^3^	
CP	17.00
NDF	36.20
ADF	21.90
EE	5.89
Ash	7.60
Ca	0.62
P	0.56
NFC	37.50
NE_L_ ^4^, Mcal/kg	1.57
Met, of the metabolizable protein	24.6

^1^ DM = dry matter; CP = crude protein; NDF = neutral detergent fiber; ADF = acid detergent fiber; EE = ether extract; NFC = non-fiber carbohydrate; NE_L_ = net energy for lactation. ^2^ Concentrate includes: 201.5 g/kg Ca (HCO_3_)_2_, 183.1 g/kg stone powder, 219.7 g/kg salt, 29.3 g/kg rumen-protected methionine, 91.6 g/kg yeast, 91.6 g/kg yeast culture, 91.6 g/kg MgO, 91.6 g/kg premix. Premix includes (per kilogram of DM): 17 KIU of vitamin D, 73 KIU of vitamin A, 1200 IU of vitamin E, 60 mg of Co, 20 mg of Se, 40 mg of Fe, 255 mg of Cu, 708 mg of Mn, 40 mg of I. ^3^ The nutrients of CP, NDF, ADF, EE, Ash, Ca and P were determined in laboratory. ^4^ Net energy for lactation, calculated based on the Ministry of Agriculture of China recommendations (NY/T 34-2004 [9]).

**Table 2:** Effects of methionine dipeptide supplementation on lactation performance of dairy cows.

Items ^3^	Treatments ^1^			*p*-Value ^2^		
	CON	MM	RPMM	Treat	Week	T × W
DMI, kg/d	24.53 ± 1.39	25.16 ± 0.68	24.84 ± 0.39	0.24	0.26	0.41
Milk yield, kg/d						
Raw	30.81 ± 0.56 ^b^	31.72 ± 1.27 ^b^	32.29 ± 0.83 ^a^	0.01	<0.01	0.17
ECM ^4^	28.74 ± 0.49 ^c^	30.23 ± 0.98 ^b^	30.61 ± 0.76 ^a^	0.04	<0.01	0.08
Fat	1.05 ± 0.03	1.08 ± 0.03	1.12 ± 0.02	0.18	0.31	0.78
Protein	1.04 ± 0.02 ^b^	1.13 ± 0.03 ^a^	1.15 ± 0.02 ^a^	0.03	<0.01	0.04
Lactose	1.64 ± 0.04	1.71 ± 0.04	1.62 ± 0.04	0.35	0.04	0.69
Milk composition, %						
Fat	3.23 ± 0.10	3.30 ± 0.09	3.30 ± 0.11	0.64	<0.01	0.26
Protein	3.35 ± 0.06 ^b^	3.48 ± 0.08 ^a^	3.50 ± 0.07 ^a^	0.04	<0.01	0.02
Lactose	5.24 ± 0.13	5.27 ± 0.14	5.17 ± 0.13	0.74	<0.01	0.71
MUN, mg/dL	12.48 ± 2.71	14.14 ± 2.73	12.30 ± 2.31	0.56	0.41	0.67
Feed efficiency ^5^	1.26 ± 0.02	1.27 ± 0.01	1.31 ± 0.02	0.16	<0.01	0.42

^1^ CON = control group; MM = methionine dipeptide; RPMM = rumen-protected methionine dipeptide. ^2^ Treat = effect of Met-Met supplementation; Week = effect of time; T × W, interaction of treat and week. ^3^ DMI = dry matter intake; ECM = energy-corrected milk; MUN = milk urea nitrogen. ^4^ ECM = 12.55 × fat yield (kg/d) + 7.39 × protein yield (kg/d) + 5.34 × lactose yield (kg/d), from Orth. ^5^ Feed efficiency = milk yield/DMI. ^a–c^ Means within a row with different superscripts differ (*p* < 0.05).

**Table 3:** Effects of methionine dipeptide supplementation on plasma amino acid concentrations of dairy cows.

Items, mg/L	Treatments ^1^			*p*-Value
	CON	MM	RPMM	
Essential amino acids				
Arginine	5.94 ± 1.34	6.78 ± 0.89	6.40 ± 1.15	0.62
Histidine	6.20 ± 1.04	6.20 ± 0.79	6.56 ± 1.06	0.88
Isoleucine	8.35 ± 1.50	7.68 ± 0.76	8.06 ± 0.98	0.76
Leucine	11.49 ± 1.90	11.10 ± 0.95	11.51 ± 1.05	0.93
Lysine	6.78 ± 0.85	7.57 ± 0.84	6.80 ± 0.90	0.54
DL-Methionine	1.52 ± 0.38	1.71 ± 0.19	1.70 ± 0.19	0.51
Phenylanaline	6.85 ± 2.29	8.18 ± 1.12	8.57 ± 1.32	0.28
Threonine	19.23 ± 2.05	22.44 ± 2.09	22.31 ± 1.77	0.19
Valine	17.08 ± 2.22	17.32 ± 1.55	17.60 ± 1.56	0.96
Nonessential amino acids				
Alanine	12.17 ± 2.21	12.38 ± 2.04	10.66 ± 1.67	0.52
Asparagine	1.01 ± 0.35	1.07 ± 0.40	0.69 ± 0.61	0.39
Cysteine	3.93 ± 0.18	4.35 ± 0.75	4.28 ± 0.46	0.65
Glutamine	8.61 ± 1.06	10.21 ± 1.67	9.24 ± 1.52	0.39
Glycine	10.65 ± 2.13	11.09 ± 0.64	12.41 ± 1.10	0.33
Proline	4.60 ± 1.00	5.62 ± 1.23	4.87 ± 0.72	0.29
Serine	4.73 ± 0.80	5.24 ± 0.37	5.33 ± 0.61	0.49
Tyrosine	10.04 ± 2.52	11.87 ± 1.58	11.11 ± 1.51	0.41

^1^ CON = control group; MM = methionine dipeptide; RPMM = rumen-protected methionine dipeptide.

**Table 4:** Effects of methionine dipeptide on rumen fermentation of dairy cows.

Items ^2^	Treatments ^1^			*p*-Value
	CON	MM	RPMM	
pH	6.01 ± 0.22	6.04 ± 0.12	6.06 ± 0.09	0.85
VFAs, mmol/L				
Total VFA	102.10 ± 7.08 ^b^	90.98 ± 3.47 ^b^	112.70 ± 4.17 ^a^	0.04
Acetate (A)	57.79 ± 1.79 ^b^	51.94 ± 2.34 ^b^	62.85 ± 2.78 ^a^	0.05
Propionate (P)	28.38 ± 4.62	26.23 ± 8.66	31.16 ± 6.45	0.34
Butyrate	12.67 ± 1.24 ^a^	10.79 ± 2.81 ^b^	14.35 ± 1.18 ^a^	<0.01
Valerate	1.23 ± 0.20 ^b^	1.38 ± 0.18 ^ab^	1.58 ± 0.22 ^a^	<0.01
Isobutyrate	0.77 ± 0.15	0.60 ± 0.24	0.80 ± 0.28	0.16
Isovalerate	1.17 ± 0.11	1.11 ± 0.38	1.32 ± 0.18	0.20
A:P ratio	2.08 ± 0.04	2.02 ± 0.02	2.05 ± 0.12	0.94
NH_3_-N, mg/dL	12.38 ± 0.64	12.24 ± 0.48	11.49 ± 0.89	0.81

^1^ CON = control group; MM = methionine dipeptide; RPMM = rumen-protected methionine dipeptide. ^2^ VFA = volatile fatty acids. ^a,b^ Means within a row with different superscripts differ (*p* < 0.05).

## Data Availability

Data are contained within the article.
